# RNA inverse folding using Monte Carlo tree search

**DOI:** 10.1186/s12859-017-1882-7

**Published:** 2017-11-06

**Authors:** Xiufeng Yang, Kazuki Yoshizoe, Akito Taneda, Koji Tsuda

**Affiliations:** 1 Department of Computational Biology and Medical Sciences, Graduate School of Frontier Sciences, The University of Tokyo, 5-1-5 Kashiwanoha, Kashiwa, 277-8561 Japan; 2 Graduate School of Science and Technology, Hirosaki University, 3 Bunkyo-cho, Hirosaki, 036-8561 Japan; 3 Center for Materials Research by Information Integration, National Institute for Materials Science, 1-2-1 Sengen, Tsukuba, 305-0047 Japan; 4 RIKEN Center for Advanced Intelligence Project, 1-4-1 Nihombashi Chuo-ku, Tokyo, 103-0027 Japan

**Keywords:** Monte Carlo tree search, RNA inverse folding, Local update, Pseudoknotted structure

## Abstract

**Background:**

Artificially synthesized RNA molecules provide important ways for creating a variety of novel functional molecules. State-of-the-art RNA inverse folding algorithms can design simple and short RNA sequences of specific GC content, that fold into the target RNA structure. However, their performance is not satisfactory in complicated cases.

**Result:**

We present a new inverse folding algorithm called MCTS-RNA, which uses Monte Carlo tree search (MCTS), a technique that has shown exceptional performance in Computer Go recently, to represent and discover the essential part of the sequence space. To obtain high accuracy, initial sequences generated by MCTS are further improved by a series of local updates. Our algorithm has an ability to control the GC content precisely and can deal with pseudoknot structures. Using common benchmark datasets for evaluation, MCTS-RNA showed a lot of promise as a standard method of RNA inverse folding.

**Conclusion:**

MCTS-RNA is available at https://github.com/tsudalab/MCTS-RNA.

**Electronic supplementary material:**

The online version of this article (doi:10.1186/s12859-017-1882-7) contains supplementary material, which is available to authorized users.

## Background

The function of RNA transcripts is tied to their three-dimensional molecular structures, itself primarily determined by secondary structures. For this reason, computational prediction of RNA secondary structure has been a popular subject of research for decades [1–5]. To obtain an RNA sequence with a desired function in synthetic biology, it is often necessary to design a functional RNA sequence whose stable structure matches a user-specified target structure. From the viewpoint of computational biology, this is exactly the inverse problem of RNA secondary structure prediction, and is called *RNA inverse folding* [4, 6, 7].

To date, RNA inverse folding approaches have been successfully applied to create RNAs that function in vitro and in vivo. Dotu et al. [8] performed RNA inverse folding of hammerhead ribozymes and experimentally validated the self-cleaving function of the designed ribozymes. Wachsmuth et al. [9] have constructed an *in silico* artificial riboswitches design pipeline in an inverse folding-like manner, which repeatedly utilized an RNA secondary structure prediction method to obtain RNA sequences that fold into specified secondary structures.

In RNA inverse folding algorithms, a reward function (or objective function) that measures the similarity between the folded RNA structure and a target structure is used to evaluate a generated RNA sequence. In addition, it takes into account other sequence properties, such as GC content (fraction of guanine and cytosine), that crucially affect the functions of RNA molecules [10].

**Fig. 1:**
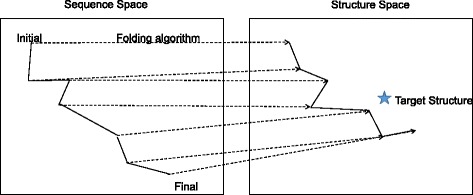
Schematic illustration of local search. Given an initial sequence (i.e., a point in the sequence space), secondary structure prediction is applied to obtain the corresponding secondary structure (i.e., a point in the structure space). Based on the difference between the predicted and target structures, the sequence is updated. After repeating the update until a termination condition is met, the best sequence is chosen from the set of generated sequences

To deal with the huge search space whose size is exponential to sequence length, a number of optimization techniques have been applied to RNA inverse folding (Table 1). Most approaches rely on heuristics such as local search [11–14], evolutionary algorithms [6, 15–17], weighted sampling [18], or ant colony optimization [7]. RNAiFold [19] uses constraint programming so that it can find all sequences matching the target structure. Local search algorithms apply update rules repeatedly to make the predicted structure as close to the target structure as possible (Fig. 1). Local search is often combined with evolutionary algorithms to improve accuracy [17, 18]. Updates are designed so that the predicted structure is improved in terms of reward.

**Table 1:** Existing tools and their ability to control GC-content and handle pseudoknot structures

Tools	Algorithm	GC content	Pseudoknot
RNAinverse [11]	Local search	No	No
RNA-SSD [12]	Stochastic local search	No	No
	and structure decomposition		
INFO-RNA [13]	Dynamic programming	No	No
	and local search		
NUPACK [14]	Minimization of ensemble	No	No
	defect and structure		
	decomposition		
RNAexinv [35]	Simulated annealing	No	No
Frnakenstein [15]	Genetic algorithm	No	No
EteRNABot [36]	Downhill simplex algorithm	No	No
ERD [16]	Evolutionary algorithm	No	No
	and structure		
	decomposition		
RNAifold [19]	Constraint programming	Yes	No
	and structure		
	decomposition		
IncaRNAtion [18]	Weighted sampling	Yes	No
	algorithm and		
	local search		
MODENA [17]	Multi-objective	Yes	Yes
	genetic algorithm		
antaRNA [7, 30]	Ant colony optimization	Yes	Yes
Enzymer [37]	Adaptive weighted	No	Yes
	sampling		
MCTS-RNA	Monte Carlo tree search	Yes	Yes

Inverse folding algorithms depend on secondary structure prediction methods such as RNAfold [4] for nested structures and pKiss [20] for pseudoknot structures. RNAifold [19], IncaRNAtion [18], MODENA [17] and antaRNA [7] design RNA sequences for nested structures with GC content control. Among them, antaRNA and MODENA allow pseudoknot target structures. To deal with pseudoknots, antaRNA uses pKiss [20] as its structure prediction method, while MODENA uses either IPknot [21] or HotKnots [22].

In this paper, we develop a new algorithm called MCTS-RNA that employs Monte Carlo tree search (MCTS) to solve the RNA inverse folding problem. MCTS is a randomized best-first search method that showed exceptional performance in computer Go [23, 24]. In addition, it has been successfully applied to computational biology [25] and other research domains [23, 26]. In an RNA sequence, each base can have a very different impact on the structure [27]. Replacement of an essential base may change the structure completely, while a non-essential base may be totally irrelevant. We employ MCTS to discover the set of essential bases that determines the secondary structure. In our analogy, base determination corresponds to placing a stone in Go. In computer Go, scoring an intermediate state, i.e., estimation of winning probabilities given a set of placed stones, is crucial to the overall performance. Likewise, we need to develop a way to evaluate a partially determined RNA sequence with respect to the possibility of creating sequences with the target structure.

**Fig. 2:**
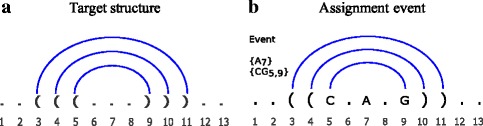
Target RNA secondary structure and assignment events. **a** This target structure of length *N*=13 has three base pairs and seven free bases. **b** After the events {*A*_7_} and {*C* *G*_5,9_}, three positions are determined

In our notation, an *event* indicates base assignment to one position or two positions at once (Fig. 2). For example, the events {*A*_7_} and {*C* *G*_5,9_} indicates that A is assigned to position 7, C and G are assigned to positions 5 and 9. Let *ℓ* denote the sum of the number of free bases and that of base pairs in the target structure. The complete search tree is defined as the tree of depth *ℓ*, where the children of a node represents all possible events. It is obviously impossible to keep the complete tree in memory. Starting from the root node alone, MCTS expands the tree gradually by identifying the most promising node and expanding its children. To evaluate a node, a full sequence (i.e. an initial sequence) is generated by randomly choosing the remaining events, which is then used as an initial point of local search. Each node has a UCB (Upper Confidence Bound) score [28] determined by the reward of the best sequence obtained by local search and the number of visits to the node. By taking the number of visits into account, our algorithm can avoid focusing too much on the same part of the search tree.

In contrast to evolutionary algorithms, MCTS has a stronger theoretical background [29]. The regret bound of the UCB score, for example, is well-studied in literature [28]. In heuristic optimization, it is essential to control the balance between exploitation and exploration [23]: This is a difficult task for the algorithms controlled by biologically inspired parameters such as pheromone or cross-over parameters. MCTS has a simpler mechanism where the balance is controlled by a hyper-parameter *C* involved in the UCB score. In general, the success of complex algorithms involving many parameters is dependent on the proper configuration of these parameters, which can lead to difficulties adapting to different problems without changing the default parameter values.

Using standard benchmark datasets, we performed extensive experimental comparisons for both nested and pseudoknotted structures. Within a time limit of ten minutes, MCTS-RNA succeeded in creating more sequences matching the target structure than MODENA, ERD and antaRNA. Notably, MCTS-RNA produced results for some difficult Rfam families where other methods could not find a matching sequence within the time limit. These promising results demonstrate the efficiency of MCTS in RNA inverse folding, and suggest a new way to design algorithms for solving combinatorial problems in computational biology.

## Method

### Reward function

In MCTS-RNA, we design a sequence whose predicted secondary structure matches the given target structure and the GC-content remains within an acceptable range of a target value *α* ^∗^. In the search process, a reward function is employed to measure how close a sequence is to the desired one. The structural distance *d* is the Hamming distance between the parentheses representation of target and predicted secondary structures. Let us denote the sequence length of the target structure by *N*, and the GC content of the generated sequence by *α*. The reward of a sequence is defined as

1 $$  r = \left\{ \begin{array}{rl} R_{GC} + \frac{N-d}{N} & \text{for} - \delta \le \alpha - \alpha^{*} \le \delta \\ \frac{N-d}{N} & \text{otherwise} \\ \end{array} \right.  $$

where *R*_*GC*_ (> 0.0) is a weight parameter and *δ* determines the allowed deviance from *α* ^∗^. If the GC content target is not available, *r*=(*N*−*d*)/*N*.

### Sequence space

The target structure (Fig. 2) determines which positions should form base pairs. In designing a sequence, such a paired position is called a *paired site*. It can be assigned only with one from the following six base pairs [*A* *U*,*U* *A*,*G* *U*,*U* *G*,*C* *G*,*G* *C*].

The remaining free positions are called *single sites*. They are not constrained and can be assigned with any base [*A*,*C*,*G*,*U*]. The event that a pair site (*i*,*j*) is assigned with a base pair *XY* is described as {*X* *Y*_*i*,*j*_}. For a single site, it is described as {*X*_*i*_}. Random assignment of a site is defined as follows. If it is a paired site, a base pair is chosen from [*A* *U*,*U* *A*,*G* *U*,*U* *G*,*C* *G*,*G* *C*] with equal probabilities. If it is a single site, a base is chosen from [*A*,*C*,*G*,*U*] with equal probabilities.

### Monte Carlo tree search

**Fig. 3:**
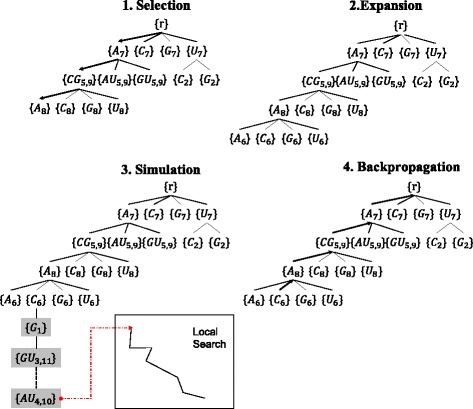
Overview of MCTS-RNA. Each node of the search tree has an assignment event. The search tree is gradually expanded by repeating the four steps: Selection, Expansion, Simulation and Backpropagation. In the selection step, the tree is traversed from the root node to a leaf node by taking the child node with the largest UCB-score at each branch. If necessary, children nodes are added to the leaf node in the expansion step. In the simulation step, a number of sequences are generated by local search. Finally, parameters at the ancestor nodes are updated in the backpropagation step. These four steps are repeated until a sequence with the target structure is found

MCTS-RNA creates a search tree where each node corresponds to an assignment event (Fig. 3). When the total number of single and pair sites is *ℓ*, the maximum depth of the tree is *ℓ*. A path from the root to a leaf represents a partially determined sequence. In the first round of MCTS-RNA, only the root node exists in the search tree. From *ℓ* sites, a site is chosen randomly. If it corresponds to a single site, four child nodes containing bases [*A*,*C*,*G*,*U*] are created under the root node. Otherwise, six nodes with base pairs [*A* *U*,*U* *A*,*G* *U*,*U* *G*,*C* *G*,*G* *C*] are created. Each node *i* contains three variables: the visit count *v*_*i*_ represents the number of visits in the search process, *z*_*i*_ denotes the immediate merit of node *i* evaluated by sequence generation, and the cumulative value *w*_*i*_ is defined as the sum of *z*_*j*_ for all descendant nodes including itself. The UCB score [28] of a node is defined as

2 $$  u_{i} = \frac{w_{i}}{v_{i}}+{C}{\sqrt{\frac{2 \ln v_{parent}}{v_{i}}}},  $$

where *C* is a constant to balance exploration and exploitation and *v*_*parent*_ is the visit count of the parent node. The variables are initialized as

3 $$  v_{i}=w_{i}=z_{i}=0, u_{i} = \infty.  $$

A round of MCTS-RNA consists of four steps: Selection, Expansion, Simulation and Backpropagation (Fig. 3). The expansion step can be skipped but the other three steps always take place. In the selection step, the tree is traversed from the root node to a leaf node by following the child with the largest UCB score *u*_*i*_. If there are ties, the winning child is chosen randomly.

If the leaf node is a rarely visited node (i.e, the visit count is smaller than the expansion threshold *β*: *v*_*i*_<*β*), the expansion step is skipped. In the simulation step, *k* sequences are generated by choosing the remaining assignment events randomly and applying *k*−1 local updates. Details of sequence generation is described in the next section. If the predicted structure of one in the *k* generated sequence is identical with the target structure, MCTS-RNA terminates immediately. Otherwise, the algorithm continues until the time limit is up. For each generated sequence, the reward function (1) is computed, and the maximum reward is stored as the immediate value *z*_*i*_. In the backpropagation step, the visit count *v*_*j*_ of each ancestor node *j* is incremented *v*_*j*_←*v*_*j*_+1 and the cumulative value is updated as *w*_*j*_←*w*_*j*_+*z*_*i*_.

If the leaf node *i* is a frequently visited node (*v*_*i*_≥*β*), the expansion step takes place. A new site is chosen randomly from the remaining sites and child nodes are created under node *i*. Similarly in the first round, four or six children are generated and initialized as (3). One child node is chosen randomly and the simulation and back propagation steps follow.

### Sequence generation by local search

In the simulation step of MCTS-RNA, we generate *k* sequences, i.e., an initial sequence and *k*−1 sequences which are obtained by progressively applying local updates to the initial sequence. The process of generating the initial sequence and local updates will keep the sites already determined by the selected path to the leaf node. We call the determined positions *essential positions*.

The initial sequence is randomly generated in such a way that the number of GCs is approximately equal to the number of desired GCs, *N* *α* ^∗^. To this aim, we repeat the following procedure until the number of GCs reachs *N* *α*∗: (i) Randomly pick up a non-essential position. (ii) If it is a paired position, choose GC or CG randomly and assign them to the paired positions; otherwise, choose G or C randomly and assign it to the position. If the number of GCs in essential positions is already larger than *N* *α*∗, the above procedure is skipped. The remaining positions are assigned with A and U in a similar manner.

In the first step of the local update, we obtain the predicted structure of the current sequence, then apply rewriting rules as many times as possible. There are two rewriting rules: (i) If two non-essential positions are paired in the target structure, but not in the predicted structure, replace them with one of [*A* *U*,*U* *A*,*C* *G*,*G* *C*] randomly. (ii) If two non-essential positions are paired in the predicted structure and not paired in the target structure, do the following:

- If they are AU or UA, replace them with AA or UU randomly.
- If they are GC or CG, replace them with CC or GG randomly.
- If they are GU or UG, replace them with one of [AC, CA, AG, GA, CU, UC] randomly.

**Fig. 4:**
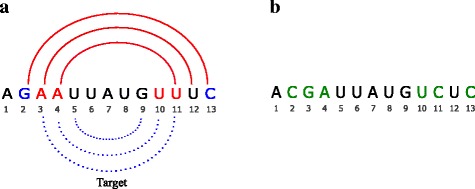
Illustration of local update. Two kinds of rewriting rules are applied to narrow the gap between predicted and target structures. Red bases {*A* *U*_3,11_} and {*A* *U*_4,10_} are updated to form base pairs, while blue bases {*G* *C*_2,13_} are updated so that the pair is destroyed. Positions 5, 7 and 9 are essential positions and not updated. **a** Nucleotides need to be updated. **b** Updated RNA sequence

The first rule is expected to form a base pair, while the second one breaks the pair. The three options in the second rule are designed to avoid changing the number of GCs in the sequence. Figure 4 shows an example of local update. Due to the first rule, {*A* *U*_3,11_} and {*A* *U*_4,10_} are updated to {*G* *C*_3,11_} and {*A* *U*_4,10_}, respectively. {*G* *C*_2,13_} is updated to {*C* *C*_2,13_} due to the second rule.

## Results and discussion

Following [6], we used 29 Rfam families as target structures to evaluate the performance of MCTS-RNA for nested structures. For pseudoknot structures, we followed [30] and used 249 structures from PseudoBase++ [31]. For nested secondary structure prediction, RNAfold was used for all the methods. For pseudoknot secondary structure prediction, IPknot and HotKnots were used for MODENA while pKiss was used for MCTS-RNA and antaRNA. MODENA has two different versions [6, 17] and the latest version was used for all the comparisons. In regard to the reward function, *R*_*GC*_ was fixed to 1 and *δ* was set to 0.01 for nested structures and 0.02 for pseudoknot structures. As shown later, this setting resulted in relatively strict control of the GC content in comparison with competing methods. If more efficiency is required, one can decrease *R*_*GC*_ or increase *δ* to relax the control. The number of local updates *k* was set to 50. In all competing methods, we employed their default parameters unless otherwise stated. Experiments were done on a CentOS 6.7 PC with 2.6 GHz CPU and 256 GB memory.

Given a target structure, the performance of an inverse folding method is measured as follows. For a nested structure, an inverse folding method is applied 50 times to the same structure with different random seeds. For a pseudoknot structure, the number of applications is reduced to 10 times due to heavy computational cost. Each run is considered as a *success*, if it could generate, within 10 min, at least one compliant sequence whose secondary structure matches perfectly with the target structure. If there is at least one success for a target structure, the structure is regarded as *solved*.

### Parameter optimization

**Fig. 5:**
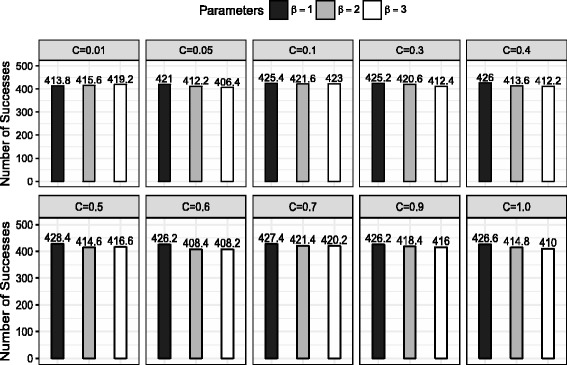
Performance of MCTS-RNA in different parameter settings. *C* is the parameter in the UCB score that determines exploration-exploitation trade-off. *β* is the expansion threshold that controls the size of the search tree. The average number of successful designs is counted for five small datasets. Each dataset consists of randomly selected 4 nested and 4 pseudoknot structures

To identify the best values of expansion threshold *β* and trade-off parameter *C*, we applied MCTS-RNA to five small datasets with different values of *β*∈{1,2,3} and *C*∈{0.01,0.05,0.1,0.3,0.4,0.5,0.6,0.7,0.9,1.0}. Each dataset consists of four nested Rfam structures and four Pseudobase++ structures, which were randomly selected. For each dataset, MCTS-RNA was performed ten times per each structure with seven different GC content values. This resulted in total 560 MCTS-RNA runs for each of five datasets. The average number of successes over the five datasets was used to measure the performance of each parameter setting. As shown in Fig. 5, *C*=0.5 and *β*=1 turned out to be the best setting. These values will be used in all remaining experiments.

### Nested structures

**Fig. 6:**
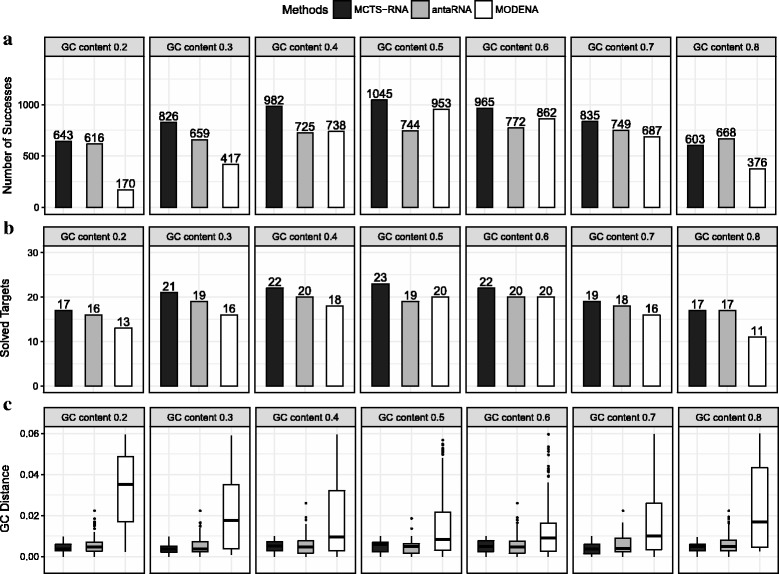
Experimental results of MCTS-RNA, antaRNA and MODENA at different target values of GC content for nested structures. **a** Total number of successful designs in 29 target structures. **b** Number of solved target structures. **c** Distribution of GC distance (i.e., the difference of obtained and target GC content)

In this experiment, MCTS-RNA is compared with existing tools with GC content control: AntaRNA and MODENA. RNAifold and IncaRNAtion are omitted, as Kleinkauf et al. [7] showed that they perform worse than antaRNA. Figures 6 a and 6 b show the total number of successes and the number of solved targets, respectively. In a realistic range of GC content, MCTS-RNA performed better than antaRNA and MODENA. At GC content 0.5, for instance, the number of successes was 40% larger than that of antaRNA. The accuracy of GC content control is shown in Fig. 6 c. MCTS-RNA and antaRNA achieved approximately the same level of accuracy, while MODENA showed significantly worse accuracy.

Table 2 shows the results for individual targets at GC content target 0.5. Tables for other target values are shown in Additional file 1: Table S8–S14. Among the structures that antaRNA failed to solve, MCTS-RNA solved 5.8S ribosomal RNA (RF00002), U1 spliceosomal RNA (RF00003), Nuclear RNase P (RF00009) and Group I catalytic intron (RF00028). Unfortunately, several difficult structures such as SNORD14 (RF00016) could not be solved by any tools.

**Table 2:** Results of MCTS-RNA, antaRNA and MODENA for individual Rfam targets

Data				MCTS-RNA		antaRNA		MODENA	
Rfam	RfamID	*N*	*ℓ*	*Sc*	*E*_*t*_	*Sc*	*E*_*t*_	*Sc*	*E*_*t*_
RF00001	5S_rRNA	117	83	44/50	196.87	4/50	28.87	0/50	–
RF00002	5_8S_rRNA|	151	127	41/50	166.06	0/50	–	13/50	64.62
RF00003	U1	161	121	5/50	371.86	0/50	–	50/50	84.43
RF00004	U2	193	149	50/50	3.4	50/50	20.04	50/50	130.99
RF00005	tRNA	74	53	50/50	0.15	50/50	0.64	50/50	32.25
RF00006	Vault	89	69	50/50	0.38	50/50	3.65	50/50	37.49
RF00007	U12	154	112	50/50	10.08	19/50	8.19	49/50	76.19
RF00008	Hammerhead_3	54	39	50/50	0.49	50/50	0.33	50/50	28.32
RF00009	RNaseP_nuc	348	293	48/50	84.58	0/50	–	0/50	–
RF00010	RNaseP_bact_a	357	255	0/50	–	0/50	–	0/50	–
RF00011	RNaseP_bact_b	382	286	0/50	–	0/50	–	0/50	–
RF00012	U3	215	176	50/50	5.64	50/50	30.6	50/50	197.66
RF00013	6S	185	137	50/50	31.05	46/50	12.83	50/50	124.12
RF00014	DsrA	87	58	50/50	0.1	44/50	0.78	42/50	40.6
RF00015	U4	140	109	50/50	2.07	22/50	10.59	49/50	62.18
RF00016	SNORD14	129	112	0/50	–	0/50	–	0/50	–
RF00017	SRP_euk_arch4	301	200	49/50	133.19	44/50	56.24	50/50	452.17
RF00018	CsrB	360	311	0/50	–	0/50	–	0/50	–
RF00019	Y_RNA	83	60	50/50	1.51	49/50	1.67	50/50	36.32
RF00020	U5	119	89	0/50	–	0/50	–	0/50	–
RF00021	Spot_42	118	81	50/50	0.26	50/50	0.98	50/50	55.34
RF00022	GcvB	148	115	50/50	1.34	49/50	10.04	50/50	74.6
RF00024	Telomerase-vert	451	346	0/50	–	0/50	–	0/50	–
RF00025	Telomerase-cil	210	173	50/50	4.88	22/50	71.32	50/50	170.58
RF00026	U6	102	97	50/50	1.6	50/50	3.37	50/50	84.66
RF00027	let-7	79	48	50/50	0.19	50/50	0.76	50/50	37.35
RF00028	Intron_gp	344	291	7/50	336.39	0/50	–	0/50	–
RF00029	Intron_gpI	73	54	50/50	2.16	14/50	7.49	50/50	35.46
RF00030	RNase_MRP	340	276	50/50	19.96	31/50	298.74	50/50	414.19
Total				1045/1450		744/1450		953/1450	

The GC content is controlled to 0.5 and the time limit is set to 10 min. *N* denotes the length of the target structure. *ℓ* describes the sum of the number of base pairs and that of free bases in the target structure. For each method, the number of successes in 50 runs is shown as *Sc*, and *E*_*t*_ indicates the average time (in seconds) required to find a compliant sequence. If no compliant sequences are found, it is left blank

To compare MCTS-RNA with ERD, we also performed experiments without GC content control. Table 3 shows that MCTS-RNA performed better than ERD and MODENA in aggregate. From a biological point of view, however, experimental results without precise GC content control may be of less importance.

**Table 3:** Experimental results of MCTS-RNA, ERD and MODENA. No GC content control is applied

Data				MCTS-RNA		ERD		MODENA	
Rfam	RfamID	*N*	*ℓ*	*Sc*	*E*_*t*_	*Sc*	*E*_*t*_	*Sc*	*E*_*t*_
RF00001	5S_rRNA	117	83	50/50	8.38	10/50	3.1	50/50	82.31
RF00002	5_8S_rRNA	151	127	32/50	88.32	12/50	3.86	20/50	93.28
RF00003	U1	161	121	48/50	83.02	0/50	–	0/50	–
RF00004	U2	193	149	50/50	1.35	21/50	2.62	50/50	138.24
RF00005	tRNA	74	53	50/50	0.3	31/50	1.35	50/50	69.39
RF00006	Vault	89	69	50/50	0.167	38/50	0.88	50/50	65.15
RF00007	U12	154	112	50/50	0.18	30/50	1.52	50/50	102.25
RF00008	Hammerhead_3	54	39	50/50	0.026	33/50	0.67	50/50	61.25
RF00009	RNaseP_nuc	348	293	23/50	61.7	32/50	19.25	0/50	–
RF00010	RNaseP_bact_a	357	255	0/50	–	0/50	–	0/50	–
RF00011	RNaseP_bact_b	382	286	0/50	–	0/50	–	0/50	–
RF00012	U3	215	176	50/50	4.08	8/50	15.3	50/50	163.32
RF00013	6S	185	137	50/50	0.6	28/50	2.43	50/50	135.10
RF00014	DsrA	87	58	50/50	0.03	32/50	0.77	50/50	73.97
RF00015	U4	140	109	50/50	0.73	25/50	1.74	50/50	88.95
RF00016	SNORD14	129	112	0/50	–	0/50	–	0/50	–
RF00017	SRP_euk_arch4	301	200	50/50	3.15	2/50	2.48	50/50	256.07
RF00018	CsrB	360	311	0/50	–	0/50	–	0/50	–
RF00019	Y_RNA	83	60	50/50	0.1	18/50	0.86	50/50	63.22
RF00020	U5	119	89	0/50	–	0/50	–	0/50	–
RF00021	Spot_42	118	81	50/50	0.06	38/50	0.83	50/50	84.99
RF00022	GcvB	148	115	50/50	1.05	31/50	1.94	50/50	97.74
RF00024	Telomerase-vert	451	346	0/50	–	0/50	–	0/50	–
RF00025	Telomerase-cil	210	173	50/50	20.23	6/50	4.59	50/50	146.33
RF00026	U6	102	97	50/50	2	50/50	0.73	50/50	65
RF00027	let-7	79	48	50/50	0.08	46/50	0.76	50/50	63.03
RF00028	Intron_gp	344	291	19/50	91.32	19/50	46.16	0/50	–
RF00029	Intron_gpI	73	54	50/50	1.2	25/50	0.79	50/50	69.92
RF00030	RNase_MRP	340	276	48/50	71.4	0/50	–	50/50	345.89
Total				1070/1450		532/1450		970/1450	

The definitions of *N*, *ℓ*, *Sc* and *E*_*t*_ are described in Table 2

### Pseudoknot structures

**Fig. 7:**
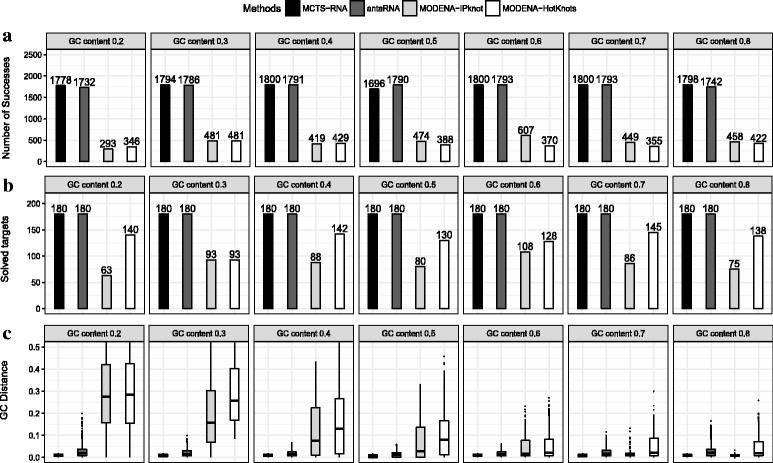
Experimental results of MCTS-RNA, antaRNA and MODENA at different target values of GC content for pseudoknot structures. **a** Total number of successfully designed sequences in 249 target structures. **b** Number of solved target structures. **c** Distribution of the error of GC content

**Fig. 8:**
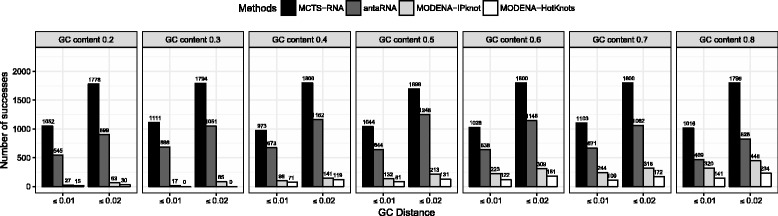
Total number of successfully designed sequences whose GC distance is within a certain threshold. As in Fig. 7, MCTS-RNA antaRNA and MODENA were applied to 249 pseudoknot structures

We applied MCTS-RNA, antaRNA and MODENA to 249 pseudoknot structures. Figure 7 shows the number of successes, the number of solved structures and the error in GC content with different GC content target values. With their default parameters, the GC content control of antaRNA was not successful in many cases. Disregarding the error in GC content, the numbers of successes found by MCTS-RNA and antaRNA were approximately the same, while MODENA showed significantly worse performance. However, when we focus on successful designs with accurate GC content, MCTS-RNA performed substantially better (Fig. 8). When the GC error is smaller than 0.01 (resp. 0.02), the number of successes of MCTS-RNA was 73% (resp. 69%) larger than that of antaRNA.

### Parameter sensitivity of antaRNA

In most literature about RNA inverse folding, software tools are evaluated with their default parameters (e.g., [7]), because users are likely to use them as they are. We nevertheless checked the performance of antaRNA when the parameters are optimized like MCTS-RNA. In optimization of antaRNA parameters, we used the same five sets of structures that were used for MCTS-RNA. The grid search was performed for three parameters *α*∈{0.2,0.5,1.0,2.0,4.0}, *β*∈{0.2,0.5,1.0,2.0,4.0},*ρ*∈{0.05,0.1,0.2}, As shown in Additional file 1: Figure S1, *α*=0.2,*β*=0.2,*ρ*=0.05 turned out to be the best. Additional file 1: Figure S2 shows the results for nested structures, where the number of successes of antaRNA increased substantially in extreme GC content settings (e.g., 0.2 and 0.8). Still, the control of GC content by antaRNA was less strict than MCTS-RNA. Additional file 1: Figure S3 shows the number of successfully designed sequences whose GC distance is smaller than 0.01. MCTS-RNA was better than antaRNA except for the case that the GC content is controlled to 0.8. In pseudoknot structures (Additional file 1: Figure S4 and S5), MCTS-RNA was consistently better than antaRNA in all GC-content settings.

### Experimental results without the structures used in parameter optimization

The accuracy of MCTS-RNA may be positively biased for the structures used in parameter optimization. In Additional file 1: Figures S6 to S9, we summarized the experimental results without the structures used in parameter optimization (Additional file 1: Table S15). Overall, we obtained similar results as in the experiments with all structures (Additional file 1: Figures S2 to S5).

### Contribution of Monte Carlo tree search

MCTS-RNA consists of MCTS and local search. In this section, we investigate how much these two parts contribute to accurate inverse folding and how they interact. For easy problems, local search from random initial sequences may suffice, but the addition of MCTS would seem necessary in difficult cases. In the following experiments, we used the 29 nested structures.

**Fig. 9:**
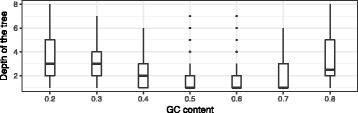
Depth of the search tree when a successfully designed sequence is found

Figure 9 shows the depth distribution of the search tree, when a compliant sequence is found, averaged over 29 Rfam structures. It is seen that, for extreme GC content targets (e.g., 0.2 and 0.8), the depth of MCTS is larger. It shows that designing sequences of medium GC content is relatively easy, so tree backtracking and expansion is not required as much.

**Fig. 10:**
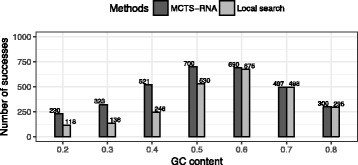
Comparison of MCTS-RNA and local search from randomly designed initial sequences. The number of RNAfold calls is fixed at 300

To measure the effect of MCTS, we compared MCTS-RNA with a simpler method of applying the local search to randomly designed initial sequences (Fig. 10). Detailed results are available in Additional file 1: Tables S1 to S7. Here, the number of local updates was constrained to 300 for both methods. No time limits were applied. The number of total successes of MCTS-RNA was about 30% larger than the local search with random initial sequences. This result indicates that the systematic search of essential bases including backtracking is necessary in RNA inverse folding.

## Conclusions

In this research work, we introduced MCTS-RNA based on Monte Carlo Tree Search to solve RNA inverse folding problem. A characteristic of this approach is that the sequence space is represented as a tree of assignment events. MCTS-RNA outperformed existing tools based on evolutionary algorithms and provided an efficient way to search in the GC-content-specific sequence space. Evolutionary algorithms keep a population of intermediate solutions and update them simultaneously. The update is designed such that a certain level of diversity is maintained to avoid falling into local minima. MCTS offers a more specific way to perform trial-and-error by setting up a search tree and allowing backtracking when the current branch turns out to be non-promising according to the UCB score.

We believe that it is easy to deploy MCTS to other real-life optimization problems, thanks to its clear separation between the problem-dependent part of the algorithm and the general search. In MCTS-RNA, the local search is the problem-dependent part, while in computer Go, it corresponds to the *playout algorithm* that randomly creates the remaining moves according to the rules of the game [24]. By contrast, in a genetic algorithm, the problem-dependent part corresponds to the definition of the gene, the rules of crossover and mutation: all aspects of the algorithm have to be calibrated to achieve top performances. Furthermore, another advantage of MCTS is that it is particularly amenable to parallelization [32]. In future work, we would like to apply MCTS to a wider range of computational biology problems such as chemical compound design [33] and discovery of diverse motifs [34].

## Additional file

Additional file 1 This supplementary report shows the experimental results for individual Rfam families where the GC content is controlled to different target values and the performance of antaRNA with optimized parameters. (PDF 1198 kb)
