# Supplementary material for: RNA inverse folding using Monte Carlo tree search
Source: BMC Bioinformatics. 2017 Nov 6;18:468. doi: 10.1186/s12859-017-1882-7 (PMC5674771; doi:10.1186/s12859-017-1882-7)
Supplement: Additional file 1 — This supplementary report shows the experimental results for individual Rfam families where the GC content is controlled to different target values and the performance of antaRNA with optimized parameters. (PDF 1198 kb) [file 12859_2017_1882_MOESM1_ESM.pdf]

RNA Inverse Folding using Monte Carlo Tree Search

Supplementary report

Xiufeng Yang[1], Kazuki Yoshizoe[4], Akito Taneda[2], Koji Tsuda[1,3,4*]

[1] Department of Computational Biology and Medical Sciences, Graduate School of Frontier Sciences, The University of Tokyo, Japan.

[2]Graduate School of Science and Technology, Hirosaki University, Japan.

[3]Center for Materials Research by Information Integration, National Institute for Materials Science, Japan.

[4]RIKEN Center for Advanced Intelligence Project, Japan.

Contents

1 Comparison results of MCTS-RNA and local search **2**

2 Comparison results of MCTS-RNA, antaRNA and MODENA **9**

2.1 Results of individual targets with different GC content values 9

2.2 Results of MCTS-RNA and antaRNA with optimized parameters 16

2.3 Results without the structures used in parameter optimization 19

1 Comparison results of MCTS-RNA and local search

Table S1 to Table S7 reports the number of successes and running time comparison of MCTS-RNA and local search with specific target GC content (0.2, 0.3, 0.4, 0.6, 0.7 0.8) on each Rfam structure. Sc represents the number of successes of 50 runs. E_t represents the average running time (in seconds) of successfully designed sequences of 50 runs.

Table S1: GC-content 0.2

Data			MCTS-RNA		Local search	
Rfam	len	ℓ	Sc	E_t	Sc	E_t
RF00001	117	83	0/50	–	0/50	–
RF00002	151	127	0/50	–	0/50	–
RF00003	16	121	0/50	–	0/50	–
RF00004	193	149	50/50	45.36	50/50	41.19
RF00005	74	53	50/50	0.85	50/50	33.02
RF00006	89	69	48/50	109.1	45/50	153.31
RF00007	154	112	13/50	276.7	25/50	229.68
RF00008	54	39	50/50	0.58	50/50	11.51
RF00009	348	293	0/50	–	0/50	–
RF00010	357	255	0/50	–	0/50	–
RF00011	382	382	0/50	–	0/50	–
RF00012	215	176	47/50	166.96	8/50	212.92
RF00013	185	137	47/50	156.23	24/50	265.88
RF00014	87	58	50/50	0.45	50/50	7.27
RF00015	140	109	5/50	504.4	1/50	52.57
RF00016	129	129	0/50	–	0/50	–
RF00017	301	200	2/50	123.05	0/50	–
RF00018	360	311	0/50	–	0/50	–
RF00019	83	60	50/50	77	44/50	145.69
RF00020	119	89	0/50	–	0/50	–
RF00021	118	81	50/50	0.45	50/50	1.46
RF00022	148	115	48/50	109.99	27/50	258.15
RF00024	451	346	0/50	–	0/50	–
RF00025	210	173	2/50	376.98	5/50	340.23
RF00026	102	97	50/50	5.8	50/50	7.32
RF00027	79	48	50/50	2.07	50/50	104.88
RF00028	344	291	0/50	–	0/50	–
RF00029	73	54	31/50	263.03	12/50	255.57
RF00030	340	276	0/50	–	0/50	–
Total			643/1450		541/1450	

Table S2: GC-content 0.3

Data			MCTS-RNA		Local search	
Rfam	len	ℓ	Sc	E_t	Sc	E_t
RF00001	117	83	0/50	–	0/50	–
RF00002	151	127	29/50	205.15	7/50	333.42
RF00003	16	121	0/50	–	0/50	–
RF00004	193	149	50/50	16.4	48/50	145.26
RF00005	74	53	50/50	1.46	50/50	6.25
RF00006	89	69	50/50	6.93	45/50	128.81
RF00007	154	112	46/50	155.91	30/50	235.55
RF00008	54	39	50/50	1.16	50/50	12.39
RF00009	348	293	1/50	400.92	1/50	255.56
RF00010	357	255	0/50	–	0/50	–
RF00011	382	382	0/50	–	0/50	–
RF00012	215	176	50/50	88.64	50/50	84.54
RF00013	185	137	50/50	66.58	34/50	290.26
RF00014	87	58	50/50	7.03	50/50	9.19
RF00015	140	109	46/50	108.15	28/50	216.14
RF00016	129	129	0/50	–	0/50	–
RF00017	301	200	5/50	295.48	5/50	216.14
RF00018	360	311	0/50	–	0/50	–
RF00019	83	60	50/50	9.89	50/50	92.02
RF00020	119	89	0/50	–	0/50	–
RF00021	118	81	50/50	0.51	50/50	6.12
RF00022	148	115	50/50	27.97	19/50	260.44
RF00024	451	346	0/50	–	0/50	–
RF00025	210	173	41/50	212.65	3/50	244.36
RF00026	102	97	50/50	1.38	50/50	0.86
RF00027	79	48	50/50	0.81	50/50	51.42
RF00028	344	291	1/50	141.66	0/50	–
RF00029	73	54	50/50	73.09	14/50	272.57
RF00030	340	276	7/50	248.83	5/50	286.79
Total			826/1450		639/1450	

Table S3: GC-content 0.4

Data			MCTS-RNA		Local search	
Rfam	len	ℓ	Sc	E_t	Sc	E_t
RF00001	117	83	7/50	123.27	2/50	294.03
RF00002	151	127	50/50	14.19	50/50	10.7
RF00003	16	121	0/50	–	1/50	177.09
RF00004	193	149	50/50	12.43	47/50	156.01
RF00005	74	53	50/50	0.4	50/50	0.69
RF00006	89	69	50/50	1.27	50/50	61.02
RF00007	154	112	50/50	15.27	46/50	219.41
RF00008	54	39	50/50	0.16	50/50	6.05
RF00009	348	293	49/50	168.69	46/50	122.49
RF00010	357	255	0/50	–	0/50	–
RF00011	382	382	0/50	–	0/50	–
RF00012	215	176	50/50	45.02	50/50	49.79
RF00013	185	137	50/50	72.95	10/50	164.29
RF00014	87	58	50/50	0.19	50/50	3.6
RF00015	140	109	50/50	12.31	44/50	190.17
RF00016	129	129	0/50	–	0/50	–
RF00017	301	200	29/50	237.53	35/50	253.78
RF00018	360	311	0/50	–	0/50	–
RF00019	83	60	50/50	2.57	50/50	24.93
RF00020	119	89	0/50	–	0/50	–
RF00021	118	81	50/50	0.38	50/50	9.42
RF00022	148	115	50/50	7.19	44/50	67.71
RF00024	451	346	0/50	–	0/50	–
RF00025	210	173	50/50	43.2	50/50	36.84
RF00026	102	97	50/50	0.86	50/50	1.49
RF00027	79	48	50/50	0.36	50/50	28.19
RF00028	344	291	14/50	285.11	17/50	220.56
RF00029	73	54	50/50	48.83	9/50	317.17
RF00030	340	276	32/50	226.45	46/50	238.95
Total			982/1450		897/1450	

Table S4: GC content 0.5

Data			MCTS-RNA		Local search	
Rfam	N	ℓ	Sc	E_t	Sc	E_t
RF00001	117	83	44/50	196.87	29/50	208.38
RF00002	151	127	41/50	166.06	49/50	181.38
RF00003	161	121	5/50	371.86	7/50	332.26
RF00004	193	149	50/50	3.4	50/50	3.44
RF00005	74	53	50/50	0.15	50/50	0.08
RF00006	89	69	50/50	0.38	50/50	0.19
RF00007	154	112	50/50	10.08	50/50	142.08
RF00008	54	39	50/50	0.49	45/50	1.33
RF00009	348	293	48/50	84.58	30/50	78.19
RF00010	357	255	0/50	–	0/50	–
RF00011	382	286	0/50	–	0/50	–
RF00012	215	176	50/50	5.64	50/50	5.39
RF00013	185	137	50/50	31.05	50/50	59.93
RF00014	87	58	50/50	0.1	50/50	0.98
RF00015	140	109	50/50	2.07	50/50	2.59
RF00016	129	112	0/50	–	0/50	–
RF00017	301	200	49/50	133.19	20/50	192.02
RF00018	360	311	0/50	–	0/50	–
RF00019	83	60	50/50	1.51	50/50	20.02
RF00020	119	89	0/50	–	0/50	–
RF00021	118	81	50/50	0.26	50/50	5.8
RF00022	148	115	50/50	1.34	50/50	1.73
RF00024	451	346	0/50	–	0/50	–
RF00025	210	173	50/50	4.88	50/50	4.06
RF00026	102	97	50/50	1.6	50/50	2.06
RF00027	79	48	50/50	0.19	50/50	11.47
RF00028	344	291	7/50	336.39	15/50	225.89
RF00029	73	54	50/50	2.16	34/50	230.82
RF00030	340	276	50/50	19.96	50/50	16.58
total			1045/1450		979/1450	

Table S5: GC-content 0.6

Data			MCTS-RNA		Local search	
Rfam	len	ℓ	Sc	E_t	Sc	E_t
RF00001	117	83	50/50	19.56	50/50	9.53
RF00002	151	127	0/50	–	2/50	305.68
RF00003	16	121	26/50	213.4	29/50	250.79
RF00004	193	149	50/50	2.31	50/50	2.43
RF00005	74	53	50/50	0.08	50/50	0.02
RF00006	89	69	50/50	0.47	50/50	0.58
RF00007	154	112	50/50	4.11	50/50	8.24
RF00008	54	39	50/50	0.27	50/50	0.3
RF00009	348	293	4/50	232.75	4/50	313.79
RF00010	357	255	0/50	–	0/50	–
RF00011	382	382	0/50	–	0/50	–
RF00012	215	176	50/50	26.39	50/50	25.7
RF00013	185	137	50/50	3.56	50/50	4.42
RF00014	87	58	50/50	0.13	50/50	2.86
RF00015	140	109	50/50	2.77	50/50	2.66
RF00016	129	129	0/50	–	0/50	–
RF00017	301	200	48/50	70.17	30/50	94.1
RF00018	360	311	0/50	–	0/50	–
RF00019	83	60	50/50	0.39	50/50	1.03
RF00020	119	89	0/50	–	0/50	–
RF00021	118	81	50/50	0.5	50/50	6.1
RF00022	148	115	50/50	1.86	50/50	2.43
RF00024	451	346	0/50	–	0/50	–
RF00025	210	173	50/50	50.25	50/50	39.54
RF00026	102	97	50/50	4.51	50/50	5.65
RF00027	79	48	50/50	0.19	50/50	7.14
RF00028	344	291	1/50	477.45	0/50	–
RF00029	73	54	50/50	1.23	50/50	0.93
RF00030	340	276	37/50	147.8	28/50	180.03
Total			965/1450		943/1450	

Table S6: GC-content 0.7

Data			MCTS-RNA		Local search	
Rfam	len	ℓ	Sc	E_t	Sc	E_t
RF00001	117	83	50/50	57.59	40/50	15.01
RF00002	151	127	0/50	–	0/50	–
RF00003	16	121	2/50	88.78	2/50	390.86
RF00004	193	149	50/50	33.89	50/50	29.45
RF00005	74	53	50/50	0.18	50/50	0.09
RF00006	89	69	50/50	11.53	50/50	7.82
RF00007	154	112	50/50	7.86	50/50	7.22
RF00008	54	39	50/50	0.1	50/50	0.08
RF00009	348	293	0/50	–	0/50	–
RF00010	357	255	0/50	–	0/50	–
RF00011	382	382	0/50	–	0/50	–
RF00012	215	176	35/50	284.5	22/50	221.2
RF00013	185	137	50/50	3.13	50/50	4.43
RF00014	87	58	50/50	0.11	50/50	0.03
RF00015	140	109	45/50	143.12	30/50	112.48
RF00016	129	129	0/50	–	0/50	–
RF00017	301	200	50/50	17	50/50	8.78
RF00018	360	311	0/50	–	0/50	–
RF00019	83	60	50/50	0.41	50/50	0.62
RF00020	119	89	0/50	–	0/50	–
RF00021	118	81	50/50	0.17	50/50	0.09
RF00022	148	115	50/50	47.97	50/50	35
RF00024	451	346	0/50	–	0/50	–
RF00025	210	173	3/50	241.36	10/50	292.16
RF00026	102	97	50/50	24.74	50/50	18.67
RF00027	79	48	50/50	0.25	50/50	2.77
RF00028	344	291	0/50	–	0/50	–
RF00029	73	54	50/50	2.67	50/50	3.08
RF00030	340	276	0/50	–	0/50	–
Total			835/1450		804/1450	

Table S7: GC-content 0.8

Data			MCTS-RNA		Local search	
Rfam	len	ℓ	Sc	E_t	Sc	E_t
RF00001	117	83	0/50	–	0/50	–
RF00002	151	127	0/50	–	0/50	–
RF00003	16	121	0/50	–	0/50	–
RF00004	193	149	18/50	265.02	29/50	207.78
RF00005	74	53	50/50	1.47	50/50	0.75
RF00006	89	69	34/50	254.58	44/50	225.87
RF00007	154	112	23/50	205.08	37/50	203.95
RF00008	54	39	50/50	0.24	50/50	0.25
RF00009	348	293	0/50	–	0/50	–
RF00010	357	255	0/50	–	0/50	–
RF00011	382	382	0/50	–	0/50	–
RF00012	215	176	2/50	121.2	4/50	258.62
RF00013	185	137	44/50	174.58	48/50	170.27
RF00014	87	58	50/50	0.11	50/50	0.06
RF00015	140	109	2/50	121.89	1/50	61.33
RF00016	129	129	0/50	–	0/50	–
RF00017	301	200	50/50	31.49	50/50	27.33
RF00018	360	311	0/50	–	0/50	–
RF00019	83	60	50/50	3.54	50/50	3.26
RF00020	119	89	0/50	–	0/50	–
RF00021	118	81	50/50	0.97	50/50	0.88
RF00022	148	115	28/50	283.21	40/50	268.01
RF00024	451	346	0/50	–	0/50	–
RF00025	210	173	3/50	20.33	0/50	–
RF00026	102	97	50/50	149.79	48/50	145.37
RF00027	79	48	50/50	0.23	50/50	3.73
RF00028	344	291	0/50	–	0/50	–
RF00029	73	54	49/50	99.3	50/50	24.34
RF00030	340	276	0/50	–	0/50	–
Total			603/1450		650/1450	

2 Comparison results of MCTS-RNA, antaRNA and MODENA

2.1 Results of individual targets with different GC content values

Table S8 to Table S14 reports the number of successes and running time comparison of MCTS-RNA, antaRNA and MODENA with specific target GC content (0.2, 0.3, 0.4, 0.6, 0.7 0.8) on each Rfam structure.

Table S8: GC-content 0.2

Data			MCTS-RNA		antaRNA		MODENA	
Rfam	len	ℓ	Sc	E_t	Sc	E_t	Sc	E_t
RF00001	117	83	0/50	–	0/50	–	0/50	–
RF00002	151	127	0/50	–	0/50	–	0/50	–
RF00003	16	121	0/50	–	0/50	–	0/50	–
RF00004	193	149	50/50	45.36	50/50	32.86	0/50	–
RF00005	74	53	50/50	0.85	50/50	1.79	18/50	34.21
RF00006	89	69	48/50	109.1	50/50	10.38	1/50	39.1
RF00007	154	112	13/50	276.7	6/50	25.46	3/50	79.86
RF00008	54	39	50/50	0.58	50/50	0.84	17/50	29.4
RF00009	348	293	0/50	–	0/50	–	0/50	–
RF00010	357	255	0/50	–	0/50	–	0/50	–
RF00011	382	382	0/50	–	0/50	–	0/50	–
RF00012	215	176	47/50	166.96	50/50	48.76	9/50	194.67
RF00013	185	137	47/50	156.23	42/50	15.67	22/50	137.46
RF00014	87	58	50/50	0.45	43/50	0.9	18/50	39.78
RF00015	140	109	5/50	504.4	6/50	128.08	0/50	–
RF00016	129	129	0/50	–	0/50	–	0/50	–
RF00017	301	200	2/50	123.05	39/50	164.6	0/50	–
RF00018	360	311	0/50	–	0/50	–	0/50	–
RF00019	83	60	50/50	77	43/50	3.68	15/50	38.38
RF00020	119	89	0/50	–	0/50	–	0/50	–
RF00021	118	81	50/50	0.45	50/50	1.88	22/50	56.34
RF00022	148	115	48/50	109.99	41/50	17.6	8/50	74.4
RF00024	451	346	0/50	–	0/50	–	0/50	–
RF00025	210	173	2/50	376.98	4/50	212.79	0/50	–
RF00026	102	97	50/50	5.8	42/50	4.34	13/50	39.75
RF00027	79	48	50/50	2.07	50/50	1.34	15/50	36.49
RF00028	344	291	0/50	–	0/50	–	0/50	–
RF00029	73	54	31/50	263.03	0/50	–	9/50	35.78
RF00030	340	276	0/50	–	0/50	–	0/50	–
Total			643/1450		616/1450		170/1450	

Table S9: GC-content 0.3

Data			MCTS-RNA		antaRNA		MODENA	
Rfam	len	ℓ	Sc	E_t	Sc	E_t	Sc	E_t
RF00001	117	83	0/50	–	0/50	–	0/50	–
RF00002	151	127	29/50	205.15	1/50	27.52	0/50	–
RF00003	16	121	0/50	–	0/50	–	0/50	–
RF00004	193	149	50/50	16.4	50/50	15.43	23/50	129.2
RF00005	74	53	50/50	1.46	50/50	0.88	22/50	32.58
RF00006	89	69	50/50	6.93	50/50	3.18	15/50	38.65
RF00007	154	112	46/50	155.91	9/50	9.39	19/50	77.47
RF00008	54	39	50/50	1.16	50/50	0.4	15/50	28.24
RF00009	348	293	1/50	400.92	0/50	–	0/50	–
RF00010	357	255	0/50	–	0/50	–	0/50	–
RF00011	382	382	0/50	–	0/50	–	0/50	–
RF00012	215	176	50/50	88.64	50/50	27.65	50/50	184.23
RF00013	185	137	50/50	66.58	42/50	9.71	32/50	123.79
RF00014	87	58	50/50	7.03	43/50	0.65	28/50	39.7
RF00015	140	109	46/50	108.15	18/50	19.5	0/50	–
RF00016	129	129	0/50	–	0/50	–	0/50	–
RF00017	301	200	5/50	295.48	35/50	68.06	10/50	465.66
RF00018	360	311	0/50	–	0/50	–	0/50	–
RF00019	83	60	50/50	9.89	42/50	2.3	25/50	37.6
RF00020	119	89	0/50	–	0/50	–	0/50	–
RF00021	118	81	50/50	0.51	50/50	1.02	37/50	54.37
RF00022	148	115	50/50	27.97	40/50	11.98	39/50	71.78
RF00024	451	346	0/50	–	0/50	–	0/50	–
RF00025	210	173	41/50	212.65	16/50	57.4	20/50	168.73
RF00026	102	97	50/50	1.38	46/50	2.94	25/50	38.41
RF00027	79	48	50/50	0.81	50/50	0.88	41/50	36.7
RF00028	344	291	1/50	141.66	0/50	–	0/50	–
RF00029	73	54	50/50	73.09	1/50	1.56	26/50	37.56
RF00030	340	276	7/50	248.83	16/50	357.5	0/50	–
Total			826/1450		659/1450		417/1450	

Table S10: GC-content 0.4

Data			MCTS-RNA		antaRNA		MODENA	
Rfam	len	ℓ	Sc	E_t	Sc	E_t	Sc	E_t
RF00001	117	83	7/50	123.27	2/50	15.53	0/50	–
RF00002	151	127	50/50	14.19	1/50	344.15	0/50	–
RF00003	16	121	0/50	–	0/50	–	0/50	–
RF00004	193	149	50/50	12.43	50/50	13.84	50/50	113.15
RF00005	74	53	50/50	0.4	50/50	0.67	49/50	32.64
RF00006	89	69	50/50	1.27	50/50	2.52	18/50	37.31
RF00007	154	112	50/50	15.27	13/50	7.69	29/50	80.85
RF00008	54	39	50/50	0.16	50/50	0.32	45/50	28.2
RF00009	348	293	49/50	168.69	1/50	309.9		
RF00010	357	255	0/50	–	0/50	–		
RF00011	382	382	0/50	–	0/50	–		
RF00012	215	176	50/50	45.02	50/50	12.09	50/50	201.62
RF00013	185	137	50/50	72.95	47/50	9.87	26/50	118.67
RF00014	87	58	50/50	0.19	45/50	0.63	50/50	39.31
RF00015	140	109	50/50	12.31	23/50	9.86	22/50	66.04
RF00016	129	129	0/50	–	0/50	–	0/50	–
RF00017	301	200	29/50	237.53	42/50	58.11	32/50	479.22
RF00018	360	311	0/50	–	0/50	–	0/50	–
RF00019	83	60	50/50	2.57	44/50	1.61	40/50	34.47
RF00020	119	89	0/50	–	0/50	–		
RF00021	118	81	50/50	0.38	50/50	1.01	50/50	55.05
RF00022	148	115	50/50	7.19	44/50	10.07	42/50	72.28
RF00024	451	346	0/50	–	0/50	–	0/50	–
RF00025	210	173	50/50	43.2	21/50	83.1	45/50	164.96
RF00026	102	97	50/50	0.86	50/50	2.98	50/50	38.38
RF00027	79	48	50/50	0.36	50/50	0.67	50/50	36.95
RF00028	344	291	14/50	285.11	0/50	–	0/50	–
RF00029	73	54	50/50	48.83	5/50	1.1	45/50	36.11
RF00030	340	276	32/50	226.45	37/50	302.78	45/50	388.17
Total			982/1450		725/1450		738/1450	

Table S11: GC content 0.5

Data			MCTS-RNA		antaRNA		MODENA	
Rfam	N	ℓ	Sc	E_t	Sc	E_t	Sc	E_t
RF00001	117	83	44/50	196.87	4/50	28.87	0/50	–
RF00002	151	127	41/50	166.06	0/50	–	13/50	64.62
RF00003	161	121	5/50	371.86	0/50	–	50/50	84.43
RF00004	193	149	50/50	3.4	50/50	20.04	50/50	130.99
RF00005	74	53	50/50	0.15	50/50	0.64	50/50	32.25
RF00006	89	69	50/50	0.38	50/50	3.65	50/50	37.49
RF00007	154	112	50/50	10.08	19/50	8.19	49/50	76.19
RF00008	54	39	50/50	0.49	50/50	0.33	50/50	28.32
RF00009	348	293	48/50	84.58	0/50	–	0/50	–
RF00010	357	255	0/50	–	0/50	–	0/50	–
RF00011	382	286	0/50	–	0/50	–	0/50	–
RF00012	215	176	50/50	5.64	50/50	30.6	50/50	197.66
RF00013	185	137	50/50	31.05	46/50	12.83	50/50	124.12
RF00014	87	58	50/50	0.1	44/50	0.78	42/50	40.6
RF00015	140	109	50/50	2.07	22/50	10.59	49/50	62.18
RF00016	129	112	0/50	–	0/50	–	0/50	–
RF00017	301	200	49/50	133.19	44/50	56.24	50/50	452.17
RF00018	360	311	0/50	–	0/50	–	0/50	–
RF00019	83	60	50/50	1.51	49/50	1.67	50/50	36.32
RF00020	119	89	0/50	–	0/50	–	0/50	
RF00021	118	81	50/50	0.26	50/50	0.98	50/50	55.34
RF00022	148	115	50/50	1.34	49/50	10.04	50/50	74.6
RF00024	451	346	0/50	–	0/50	–	0/50	–
RF00025	210	173	50/50	4.88	22/50	71.32	50/50	170.58
RF00026	102	97	50/50	1.6	50/50	3.37	50/50	84.66
RF00027	79	48	50/50	0.19	50/50	0.76	50/50	37.35
RF00028	344	291	7/50	336.39	0/50	–	0/50	–
RF00029	73	54	50/50	2.16	14/50	7.49	50/50	35.46
RF00030	340	276	50/50	19.96	31/50	298.74	50/50	414.19
total			1045/1450		744/1450		953/1450	

Table S12: GC-content 0.6

Data			MCTS-RNA		antaRNA		MODENA	
Rfam	len	ℓ	Sc	E_t	Sc	E_t	Sc	E_t
RF00001	117	83	50/50	19.56	8/50	19.96	49/50	51.82
RF00002	151	127	0/50	–	0/50	–	4/50	67.31
RF00003	16	121	26/50	213.4	0/50	–	0/50	–
RF00004	193	149	50/50	2.31	50/50	19.05	50/50	127.04
RF00005	74	53	50/50	0.08	50/50	0.62	50/50	33.79
RF00006	89	69	50/50	0.47	50/50	3.51	46/50	36.06
RF00007	154	112	50/50	4.11	13/50	9.08	50/50	82.69
RF00008	54	39	50/50	0.27	50/50	0.36	50/50	28.59
RF00009	348	293	4/50	232.75	0/50	–	0/50	–
RF00010	357	255	0/50	–	0/50	–	0/50	–
RF00011	382	382	0/50	–	0/50	–		
RF00012	215	176	50/50	26.39	50/50	29.37	50/50	185.48
RF00013	185	137	50/50	3.56	50/50	10.69	50/50	125.43
RF00014	87	58	50/50	0.13	48/50	0.6	50/50	39.75
RF00015	140	109	50/50	2.77	34/50	12.2	50/50	62.58
RF00016	129	129	0/50	–	0/50	–	0/50	–
RF00017	301	200	48/50	70.17	46/50	50.13	50/50	467.26
RF00018	360	311	0/50	–	0/50	–	0/50	–
RF00019	83	60	50/50	0.39	49/50	1.5	50/50	36.35
RF00020	119	89	0/50	–	0/50	–	0/50	–
RF00021	118	81	50/50	0.5	50/50	1.37	49/50	55.37
RF00022	148	115	50/50	1.86	49/50	12.46	50/50	72.01
RF00024	451	346	0/50	–	0/50	–	0/50	–
RF00025	210	173	50/50	50.25	26/50	78.35	21/50	163.39
RF00026	102	97	50/50	4.51	49/50	4.37	38/50	36.69
RF00027	79	48	50/50	0.19	50/50	0.65	50/50	36.71
RF00028	344	291	1/50	477.45	1/50	280.54	0/50	–
RF00029	73	54	50/50	1.23	17/50	2.22	50/50	33.37
RF00030	340	276	37/50	147.8	32/50	270.87	5/50	428.34
Total			965/1450		772/1450		862/1450	

Table S13: GC-content 0.7

Data			MCTS-RNA		antaRNA		MODENA	
Rfam	len	ℓ	Sc	E_t	Sc	E_t	Sc	E_t
RF00001	117	83	50/50	57.59	6/50	26.28	0/50	–
RF00002	151	127	0/50	–	0/50	–	0/50	–
RF00003	16	121	2/50	88.78	0/50	–	0/50	–
RF00004	193	149	50/50	33.89	50/50	27.8	50/50	125.92
RF00005	74	53	50/50	0.18	50/50	0.97	50/50	32.89
RF00006	89	69	50/50	11.53	50/50	4.62	50/50	38.07
RF00007	154	112	50/50	7.86	11/50	65.33	49/50	78.06
RF00008	54	39	50/50	0.1	50/50	0.43	50/50	17.96
RF00009	348	293	0/50	–	0/50	–	0/50	–
RF00010	357	255	0/50	–	0/50	–	0/50	–
RF00011	382	382	0/50	–	0/50	–	0/50	–
RF00012	215	176	35/50	284.5	50/50	41.39	12/50	205.35
RF00013	185	137	50/50	3.13	48/50	16.14	49/50	129.32
RF00014	87	58	50/50	0.11	48/50	0.77	49/50	39.53
RF00015	140	109	45/50	143.12	32/50	22.67	48/50	62.54
RF00016	129	129	0/50	–	0/50	–	0/50	–
RF00017	301	200	50/50	17	50/50	58.63	34/50	486.18
RF00018	360	311	0/50	–	0/50	–	0/50	–
RF00019	83	60	50/50	0.41	49/50	1.92	50/50	35.26
RF00020	119	89	0/50	–	0/50	–	0/50	–
RF00021	118	81	50/50	0.17	50/50	2.19	50/50	55.97
RF00022	148	115	50/50	47.97	49/50	18.4	34/50	75.66
RF00024	451	346	0/50	–	0/50	–	0/50	–
RF00025	210	173	3/50	241.36	29/50	190.48	0/50	–
RF00026	102	97	50/50	24.74	46/50	7.8	12/50	38.77
RF00027	79	48	50/50	0.25	50/50	0.81	50/50	35.52
RF00028	344	291	0/50	–	0/50	–	0/50	–
RF00029	73	54	50/50	2.67	28/50	2.38	50/50	33.62
RF00030	340	276	0/50	–	0/50	–	0/50	–
Total			835/1450		749/1450		687/1450	

Table S14: GC-content 0.8

Data			MCTS-RNA		antaRNA		MODENA	
Rfam	len	ℓ	Sc	E_t	Sc	E_t	Sc	E_t
RF00001	117	83	0/50	–	6/50	188.5	0/50	–
RF00002	151	127	0/50	–	0/50	–	0/50	–
RF00003	16	121	0/50	–	0/50	–	0/50	–
RF00004	193	149	18/50	265.02	47/50	193.23	17/50	147.89
RF00005	74	53	50/50	1.47	50/50	1.76	25/50	33.73
RF00006	89	69	34/50	254.58	50/50	9.65	23/50	38.31
RF00007	154	112	23/50	205.08	22/50	38.9	17/50	81.92
RF00008	54	39	50/50	0.24	50/50	0.62	50/50	28.81
RF00009	348	293	0/50	–	0/50	–	0/50	–
RF00010	357	255	0/50	–	0/50	–	0/50	–
RF00011	382	382	0/50	–	0/50	–	0/50	–
RF00012	215	176	2/50	121.2	16/50	287.75	0/50	–
RF00013	185	137	44/50	174.58	49/50	26.19	0/50	–
RF00014	87	58	50/50	0.11	50/50	1.01	50/50	40.62
RF00015	140	109	2/50	121.89	0/50	–	0/50	–
RF00016	129	129	0/50	–	0/50	–	0/50	–
RF00017	301	200	50/50	31.49	49/50	128.82	0/50	–
RF00018	360	311	0/50	–	0/50	–	0/50	–
RF00019	83	60	50/50	3.54	49/50	3.43	36/50	38.42
RF00020	119	89	0/50	–	0/50	–	0/50	–
RF00021	118	81	50/50	0.97	50/50	3.42	50/50	57.13
RF00022	148	115	28/50	283.21	50/50	108.44	7/50	76.22
RF00024	451	346	0/50	–	0/50	–	0/50	–
RF00025	210	173	3/50	20.33	4/50	212.79	0/50	–
RF00026	102	97	50/50	149.79	47/50	44.91	0/50	–
RF00027	79	48	50/50	0.23	50/50	1.11	50/50	36.87
RF00028	344	291	0/50	–	0/50	–	0/50	–
RF00029	73	54	49/50	99.3	33/50	1.48	9/50	35.34
RF00030	340	276	0/50	–	0/50	–	0/50	–
Total			603/1450		668/1450		376/1450	

2.2 Results of MCTS-RNA and antaRNA with optimized parameters

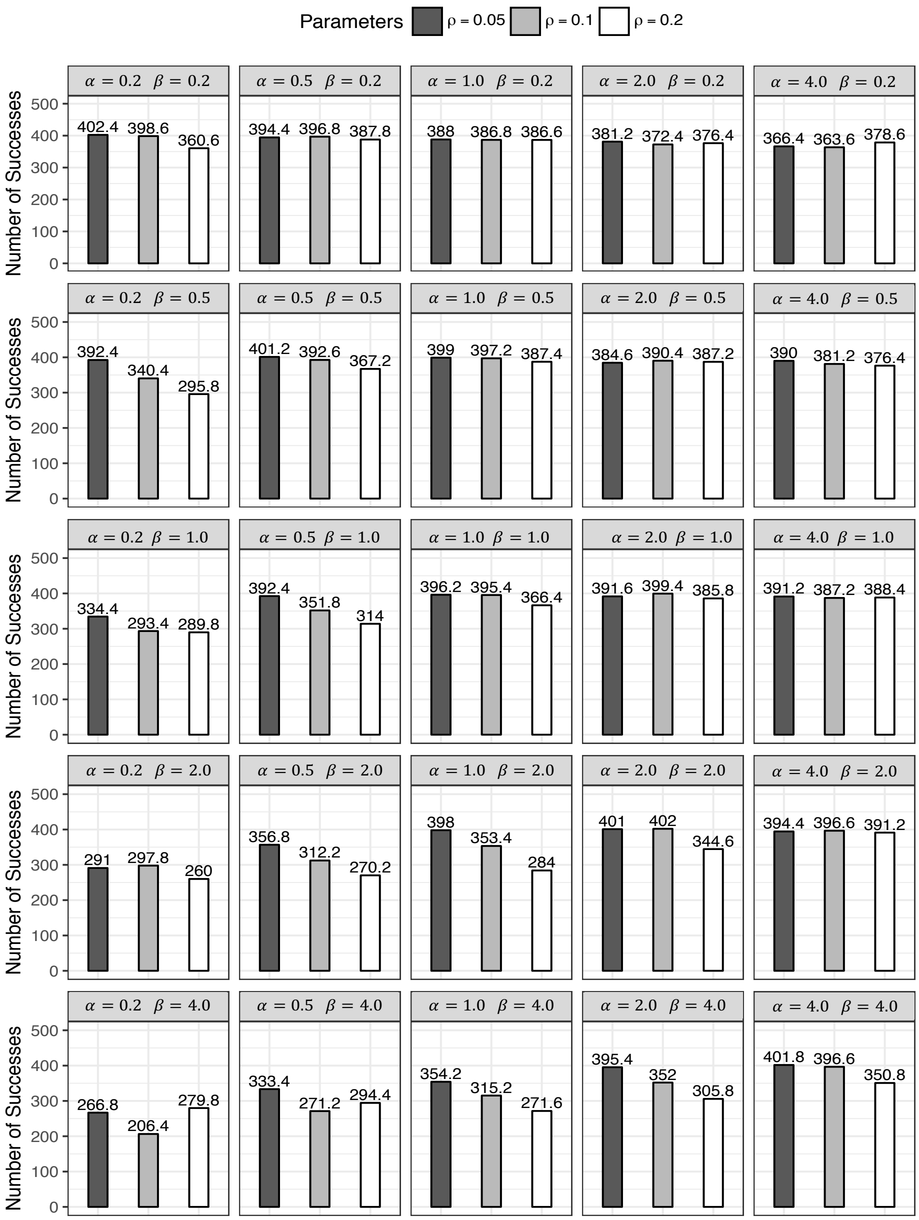

Figure S1: Performance of antaRNA at different parameter settings. α is the probability weight for terrain pheromone influence. β is the probability weight for terrain path influence. ρ is the pheromone evaporation rate (?). The average number of successes is counted for five datasets. Each dataset consists of randomly selected 4 nested and 4 pseudoknot structures. $\alpha = 0.2, \beta = 0.2, \rho = 0.05$ is the best setting and used for all the following experiments.

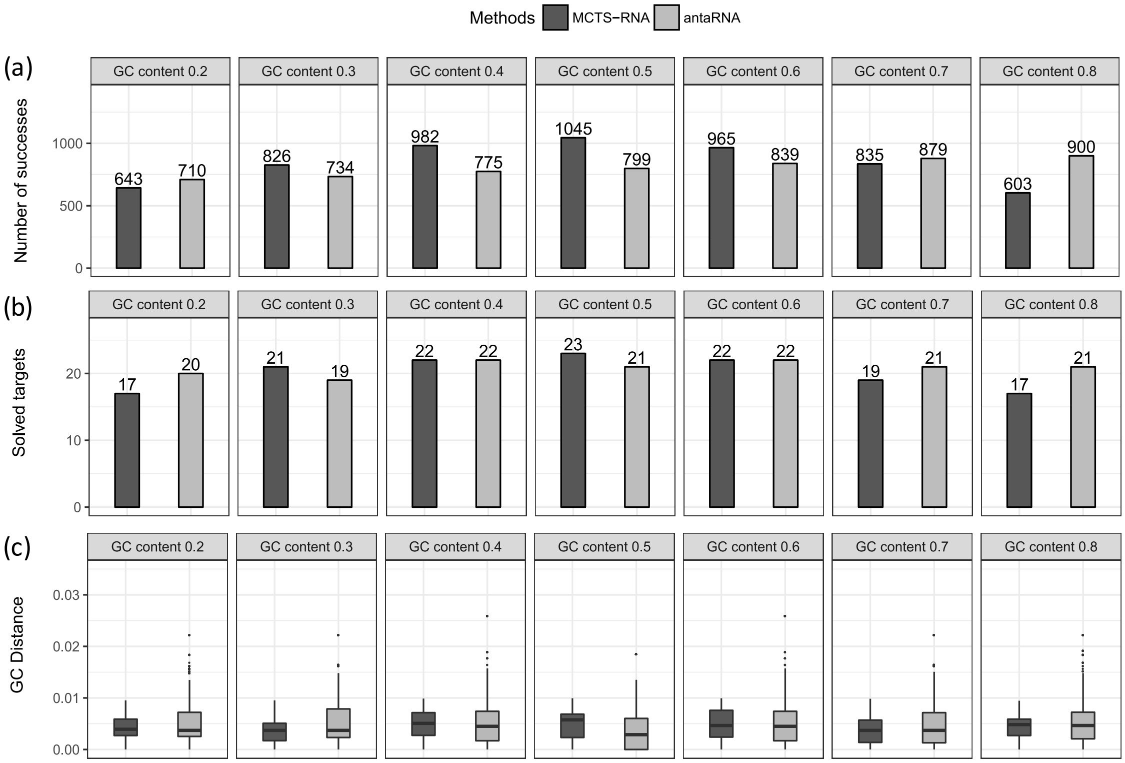

Figure S2: Experimental results of MCTS-RNA and antaRNA with optimized parameters ($\alpha = 0.2, \beta = 0.2, \rho = 0.05$) at different target values of GC content for nested structures. (a) Total number of successful designs in 29 target structures. (b) Number of solved target structures. (c) Distribution of GC distance (i.e., the difference of obtained and target GC content).

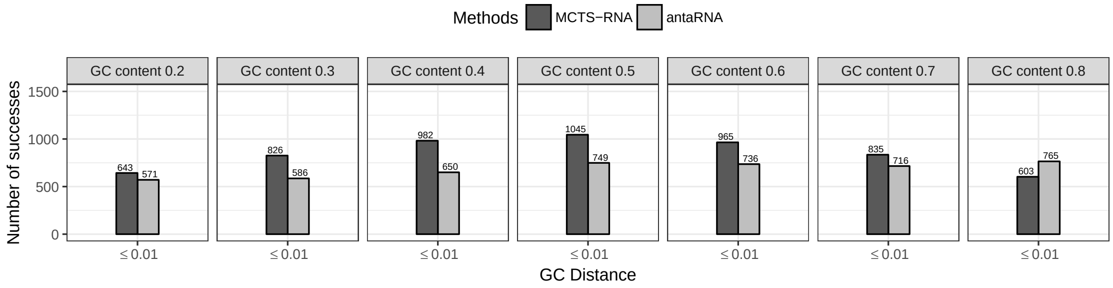

Figure S3: Total number of successfully designed sequences whose GC distance is within a certain threshold. As in Figure S2, MCTS-RNA and antaRNA with optimized parameters ($\alpha = 0.2, \beta = 0.2, \rho = 0.05$) were applied to 29 nested structures.

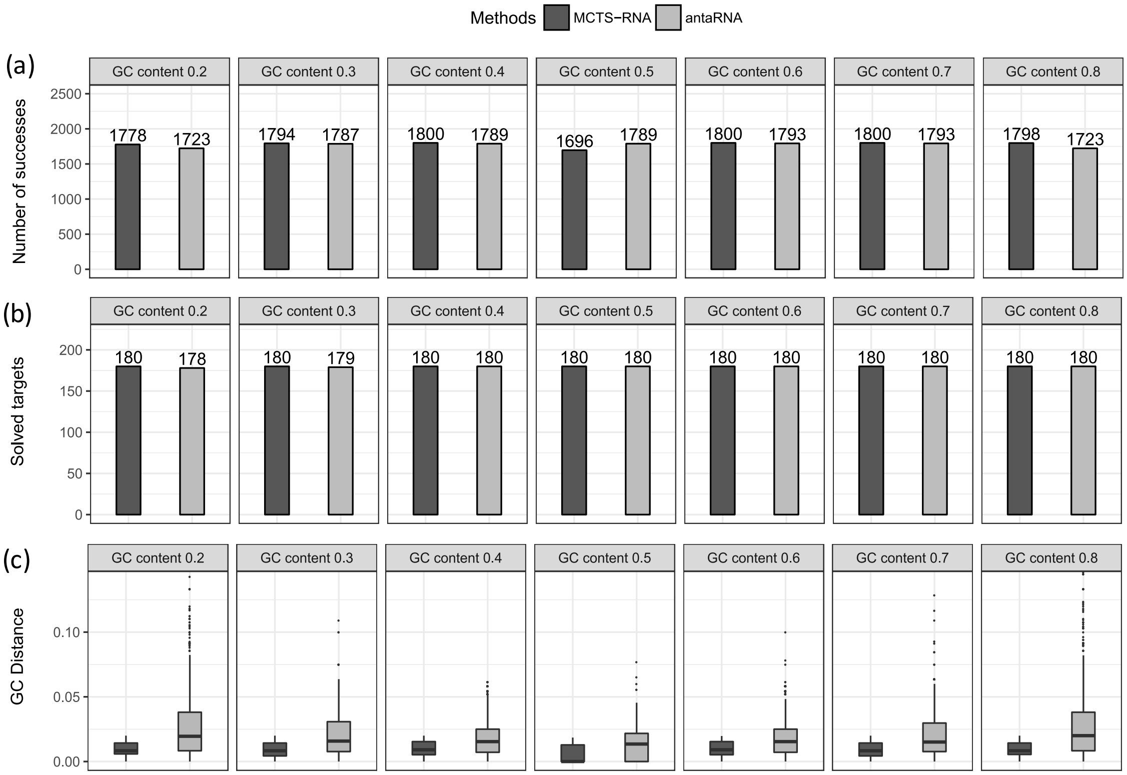

Figure S4: Experimental results of MCTS-RNA and antaRNA with optimized parameters ($\alpha = 0.2, \beta = 0.2, \rho = 0.05$) at different target values of GC content for pseudoknot structures. (a) Total number of successfully designed sequences in 249 target structures. (b) Number of solved target structures. (c) Distribution of the error of GC content.

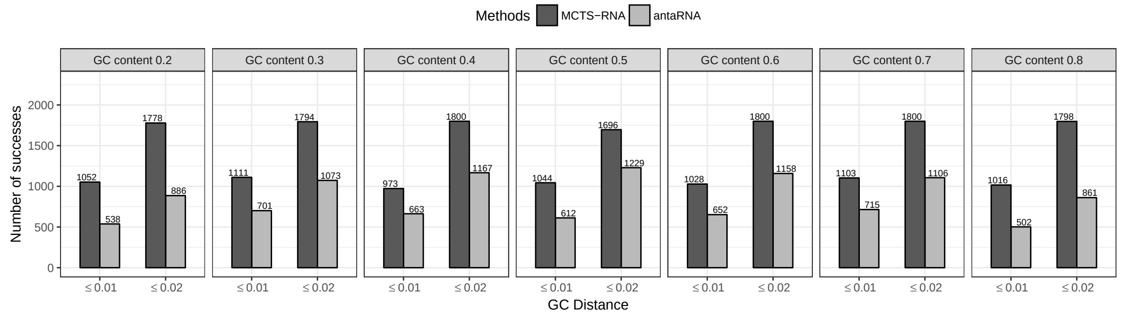

Figure S5: Total number of successfully designed sequences whose GC distance is within a certain threshold. As in Figure S3, MCTS-RNA and antaRNA with optimized parameters ($\alpha = 0.2, \beta = 0.2, \rho = 0.05$) were applied to 249 pseudoknot structures.

2.3 Results without the structures used in parameter optimization

The randomly selected five datasets (each contains randomly selected 4 nested and 4 pseudoknot structures) used for parameter optimization are shown in Table S15. After removing them, there remain 16 Rfam structures and 229 pseudobase++ structures. The experimental results using the remaining structures are shown in Figure S6-Figure S9

Table S15: Randomly selected structures for parameter optimization.

Data	Nested	Pseudoknot
Dataset1	RF00022, RF00009, RF00015, RF00010	PKB14, PKB233, PKB66, PKB70
Dataset2	RF00009, RF00019, RF00014, RF00022	PKB294, PKB298, PKB297, PKB220
Dataset3	RF00016, RF00025, RF00019, RF00029	PKB15, PKB302, PKB103, PKB286
Dataset4	RF00005, RF00014, RF00017, RF00019	PKB31, PKB8, PKB14, PKB40
Dataset5	RF00006, RF00008, RF00019, RF00005	PKB30, PKB74, PKB39, PKB41

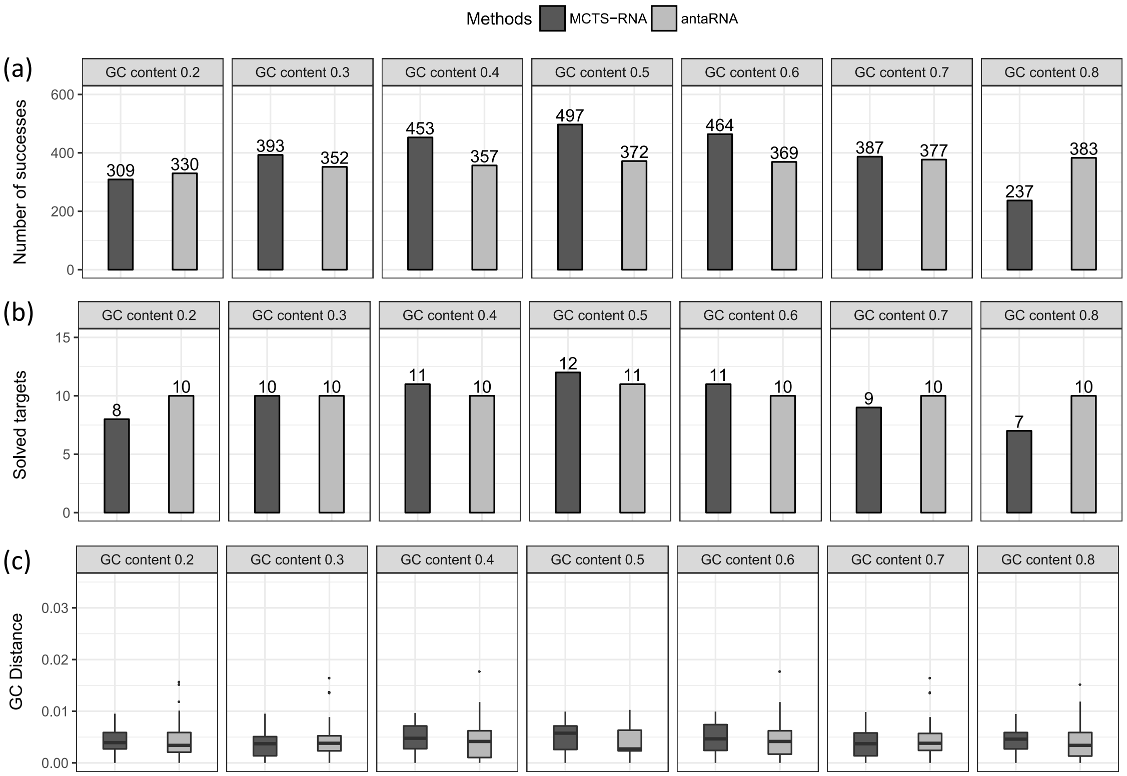

Figure S6: Experimental results of MCTS-RNA and antaRNA with optimized parameters ($\alpha = 0.2, \beta = 0.2, \rho = 0.05$) at different target values of GC content for 16 test nested structures. (a) Total number of successfully designed sequences in 16 target structures. (b) Number of solved target structures. (c) Distribution of the error of GC content.

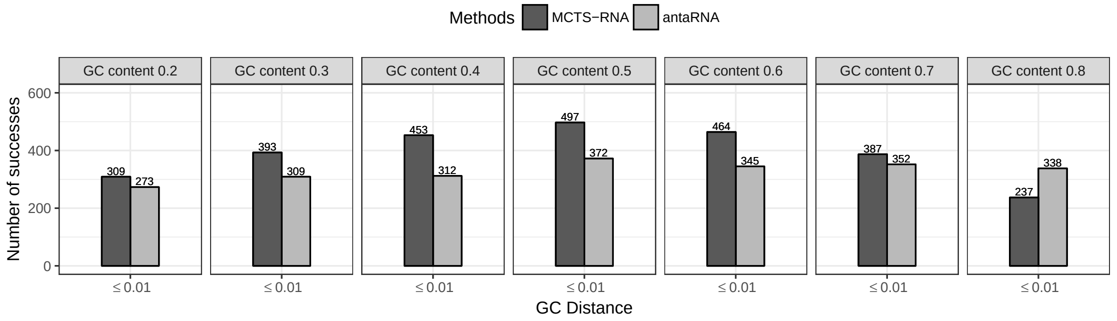

Figure S7: Total number of successfully designed sequences whose GC distance is within a certain threshold. As in Figure S6, MCTS-RNA and antaRNA with optimized parameters $(\alpha = 0.2, \beta = 0.2, \rho = 0.05)$ were applied to 16 test nested structures.

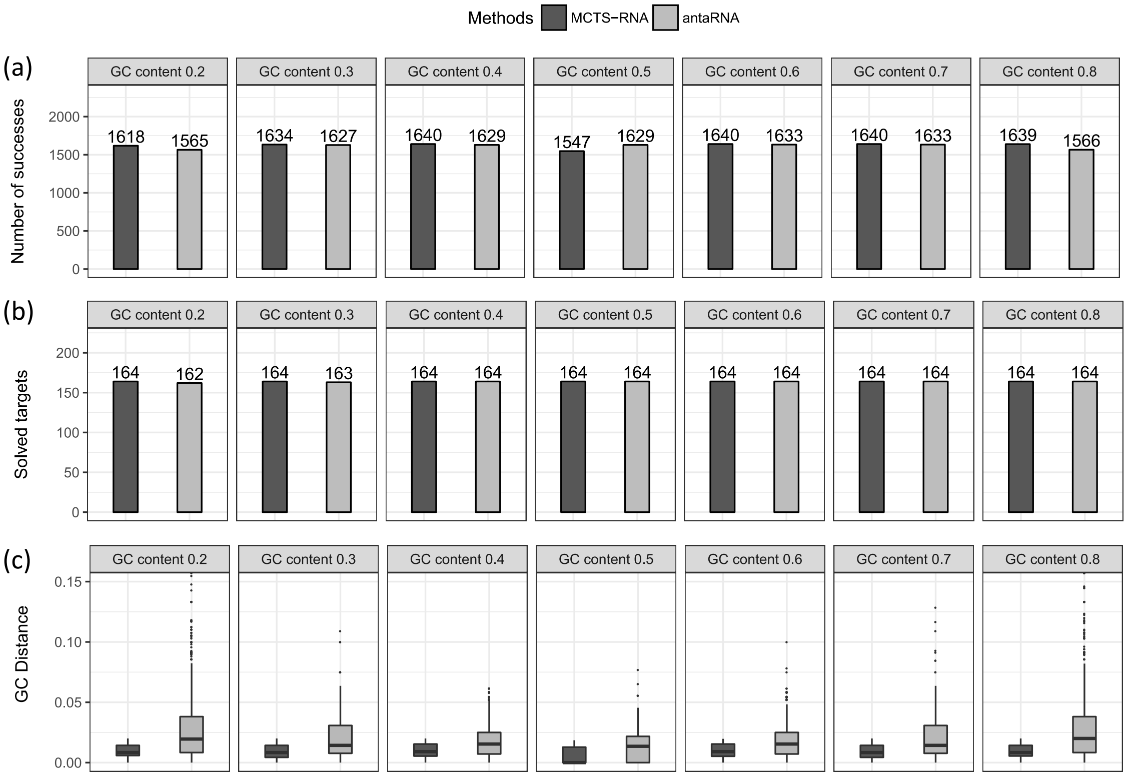

Figure S8: Experimental results of MCTS-RNA and antaRNA with optimized parameters $(\alpha = 0.2, \beta = 0.2, \rho = 0.05)$ at different target values of GC content for 229 test pseudoknot structures. (a) Total number of successfully designed sequences in 229 test target structures. (b) Number of solved target structures. (c) Distribution of the error of GC content.

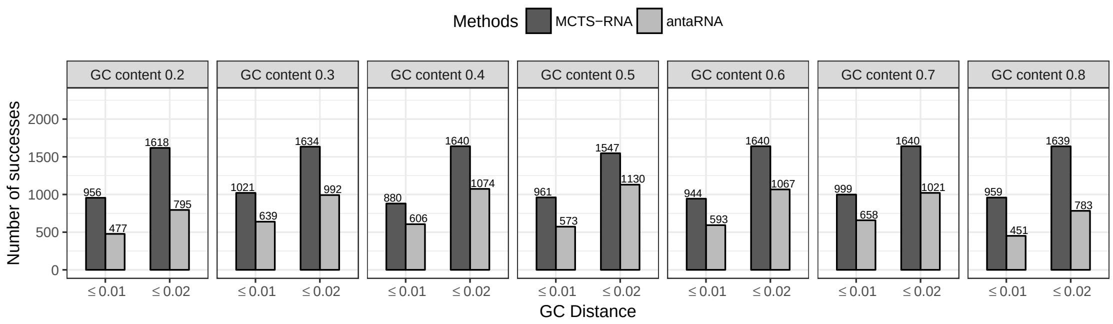

Figure S9: Total number of successfully designed sequences whose GC distance is within a certain threshold. As in Figure S8, MCTS-RNA and antaRNA with optimized parameters ($\alpha = 0.2, \beta = 0.2, \rho = 0.05$) were applied to 229 test pseudoknot structures.
